# Comparing inpatient management of chronic pelvic pain flares before and after the COVID-19 pandemic

**DOI:** 10.1530/RAF-23-0004

**Published:** 2023-06-16

**Authors:** Kevin KW Kuan, Aileen R Neilson, Andrew W Horne, Lucy HR Whitaker

**Affiliations:** 1 Edinburgh Medical School, University of Edinburgh, Edinburgh, UK; 2 Usher Institute, Edinburgh Clinical Trials Unit, University of Edinburgh, Edinburgh, UK; 3 MRC Centre for Reproductive Health, University of Edinburgh, Edinburgh, UK

**Keywords:** chronic pelvic pain, endometriosis, COVID-19, pain management, pain flare, acute admissions

## Abstract

**Graphical abstract:**

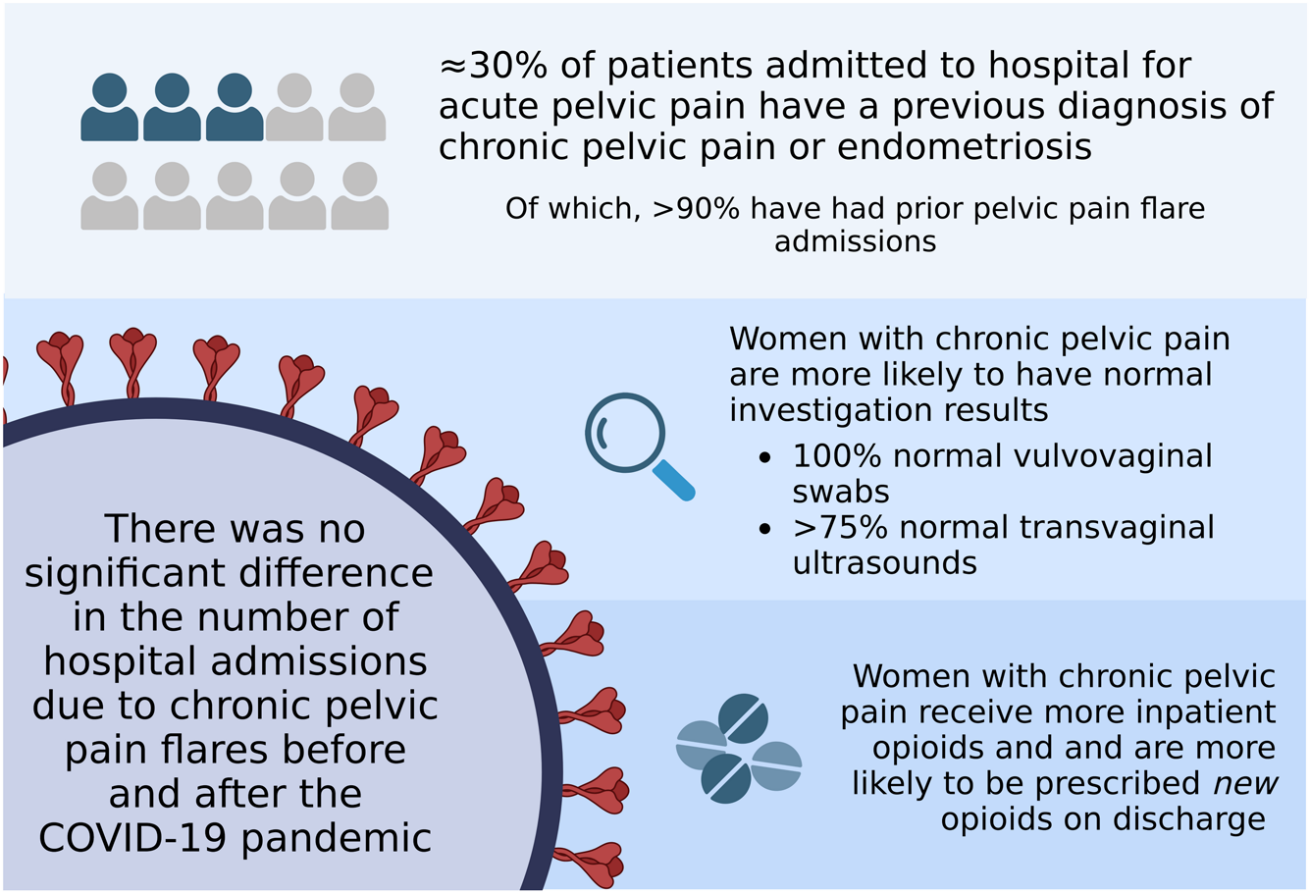

**Abstract:**

Patients with chronic pelvic pain (CPP) may experience pain exacerbations requiring hospital admissions. Due to the effects of backlogged elective surgeries and outpatient gynaecology appointments resulting from the COVID-19 pandemic, we hypothesised that there would be an increased number of women admitted with CPP flares. We conducted a retrospective review of all acute gynaecology admissions at the Royal Infirmary of Edinburgh from July to December 2018 (pre-COVID) and 2021 (post-COVID lockdown). We collected information on the proportion of emergency admissions due to CPP, inpatient investigations and subsequent management. Average total indicative hospital inpatient costs for women with CPP were calculated using NHS National Cost Collection data guidance. There was no significant difference in the number of emergency admissions due to pelvic pain before (153/507) and after (160/461) the COVID-19 pandemic. As high as 33 and 31% had a background history of CPP, respectively. Across both timepoints, investigations in women with CPP had low diagnostic yield: <25% had abnormal imaging findings and 0% had positive vaginal swab cultures. Women with CPP received significantly more inpatient morphine, pain team reviews and were more likely to be discharged with strong opioids. Total yearly inpatient costs were £170,104 and £179,156 in 2018 and 2021, respectively. Overall, emergency admission rates for managing CPP flares was similar before and after the COVID-19 pandemic. Inpatient resource use for women with CPP remains high, investigations have low diagnostic yield and frequent instigation of opiates on discharge may risk dependence. Improved community care of CPP is needed to reduce emergency gynaecology resource utilisation.

**Lay summary:**

Existing treatments for chronic pelvic pain (CPP) and endometriosis focus on surgery or hormone medication, but these are often ineffective or associated with unacceptable side-effects. As a result, women continue to experience chronic pain and often have ‘flares’ of worsening pain that may lead to hospital admission. The COVID-19 pandemic resulted in backlogged gynaecology clinics and surgeries. The aim of this study was to compare the management of emergency pelvic pain admissions for women with CPP before and after COVID-19. We also aimed to better understand their in-hospital management and estimate their hospital length of stay costs. We did not find an increase in CPP patients admitted for pelvic pain flares after the COVID-19 lockdown. Women with CPP often undergo multiple hospital tests and are often prescribed with strong pain medications which can cause long-term problems. Efforts are needed to improve long-term pain management for women with CPP.

## Introduction

Chronic pelvic pain (CPP) is characterised by persistent lower abdominal/pelvic pain lasting greater than 6 months. Up to 26% of the female population is affected by this condition which can negatively impair a patient’s physical and psychological well-being (Lamvu *et al.* 2021). The term CPP can encompass a wide variety of symptoms including, but not limited to dysmenorrhoea, non-cyclical pelvic pain, dyspareunia, fatigue and poor quality of life.

CPP can be associated with specific gynaecological conditions, such as endometriosis or adenomyosis, affecting approximately 40% of women with CPP. It is also associated with non-gynaecological conditions such as irritable bowel syndrome (IBS), interstitial cystitis or musculoskeletal problems. However, up to 55% of women with CPP have no obvious underlying pathology (Zondervan *et al.* 2018, Lamvu *et al.* 2021). Although CPP is classically considered a chronic condition, patients may experience acute pelvic pain ‘flares’ uncontrolled by their usual analgesia requiring emergency gynaecology admissions for pain relief (Agarwal *et al.* 2020, Quesada *et al.* 2022). Unfortunately, managing CPP and endometriosis remains an ongoing challenge as there is a poor understanding of the underlying pathophysiology, difficulty in diagnosis and uncertainty of treatment effectiveness (Zondervan *et al.* 2018, Horne & Missmer 2022). Women experiencing these flares often present to unscheduled care services and may require admission for investigation and pain management. Data regarding unscheduled healthcare resource usage by women with a diagnosis of CPP are predominantly limited to those with associated endometriosis (Soliman *et al.* 2016).

In March 2020, the World Health Organization officially declared COVID-19 to be a pandemic (https://www.who.int/director-general/speeches/detail/who-director-general-s-opening-remarks-at-the-media-briefing-on-covid-19---11-march-2020). Following this announcement, Scotland initiated a national lockdown and outpatient clinics and non-urgent elective surgeries for benign conditions across all specialities were postponed creating a backlog of appointments (https://www.gov.scot/news/nhs-scotland-placed-on-emergency-footing/,Carr *et al.* 2021, Bottle *et al.* 2022). In July 2020, the Scottish Government announced a gradual phase-out of lockdown restrictions and resumption of healthcare services. However, a later surge in COVID-19 cases resulted in another national lockdown on January 5, 2021, in efforts to reduce viral transmission (https://www.gov.scot/news/scotland-in-lockdown/). As well as reducing access to treatments for condition and symptom control, the restrictions on appointments created an environment of uncertainty and stress which may have contributed to the worsening of symptoms for women with CPP (Leonardi *et al.* 2020*a*, Demetriou *et al.* 2022). Consequently, this backlog may have increased the number of patients experiencing pelvic pain exacerbations due to poorer pain control following the national lockdowns.

The aim of this study was to compare inpatient management of pain flares in women with a diagnosis of CPP before and after the COVID-19 lockdown at a tertiary gynaecology unit. A cost analysis was also undertaken to quantify the NHS hospital healthcare resource use with a focus on emergency CPP admissions and the costs of the related length of stay.

## Materials and methods

All emergency gynaecology admissions due to pelvic pain at the Royal Infirmary of Edinburgh from July to December in 2018 (pre-COVID) and 2021 (post-COVID lockdown) were retrospectively reviewed via interrogation of electronic patient records. July to December in 2021 was chosen as the ‘post-COVID lockdown’ period as the second national lockdown’s restrictions were eased in May 2021 (https://www.gov.scot/news/next-steps-out-of-lockdown/). Women admitted with acute pelvic pain were included in the study and outcome variables were extracted. Women with documented CPP confirmed endometriosis either by imaging or surgery, and those with CPP under investigation were all considered to be part of the CPP cohort. Patients were excluded if they were admitted for removal of catheter or had symptoms related to oncology, pregnancy (ectopic pregnancy, miscarriage, termination of pregnancy, pregnancy symptoms), vulval conditions (Bartholin’s cyst, labial abscess, vulval abscess), heavy menstrual bleeding or post-operative complications.

### Outcomes

The primary objective of this study was to understand the absolute number and proportion of emergency admissions due to CPP flares. The secondary objectives were to compare inpatient management between women with a diagnosis of CPP vs women without CPP admitted for acute pelvic pain. Inpatient management variables include:

- Types of investigations and proportions received (full blood count (FBC), C-reactive protein (CRP), blood cultures, mid-stream urine (MSU) sample, dual vulvovaginal swab (VVS) for chlamydia/gonorrhoea NAAT and microscopy, culture and sensitivities of a high vaginal swab, pelvic ultrasound, computerised tomography (CT) and magnetic resonance imaging (MRI))
- Diagnostic yield of investigations (percentage of investigations yielding new/abnormal results)
- Inpatient management (analgesia, antibiotics, emergency surgery, pain team review)
- Diagnosis on discharge
- Discharge pain medication
- Number of days spent in hospital

### Cost analysis for women with a diagnosis of CPP

A simple-costing exercise was performed to calculate the indicative NHS hospital inpatient costs associated with managing women with a diagnosis of CPP from July to December 2018 and similarly from July to December 2021. We made the simplifying assumption of doubling these six-month cost estimates to approximate the equivalent total annual costs. This included the costs of overall hospital stay and investigation-related costs. To calculate the costs of performing specific investigations, the number of patients receiving each investigation was multiplied by the laboratory costs per unit provided by the Official Costings from Medicine & Associated Services Clinical Team, Royal Infirmary of Edinburgh. The length of hospital stay was calculated as the discharge date subtracted by the admission date. According to 2020/2021 NHS Cost Collection, the costs of inpatient hospital stay per day for non-malignant gynaecological disorders were £2263.07 (HRG code ‘MB09C’, with CC Score 0–2) with interventions and £707.66 (HRG code ‘MB09F’, with CC Score 0–2) if no interventions were required (https://www.england.nhs.uk/costing-in-the-nhs/national-cost-collection/). These price weights were assumed and applied to value the resource use associated with emergency admissions length of stay.

### Statistical analysis

Statistical analysis was performed using Version 2.3.12 of the Jamovi software programme (https://www.jamovi.org). Continuous variables were compared using the Student’s *t*-test. Categorical variables were compared using the –X^2^ test or Fisher’s exact test (for small case counts less than 5). Statistical significance was set at *P* < 0.01 to reduce the risk of false positives since multiple analyses were conducted on the same group of patients.

## Results

**Figure 1:**
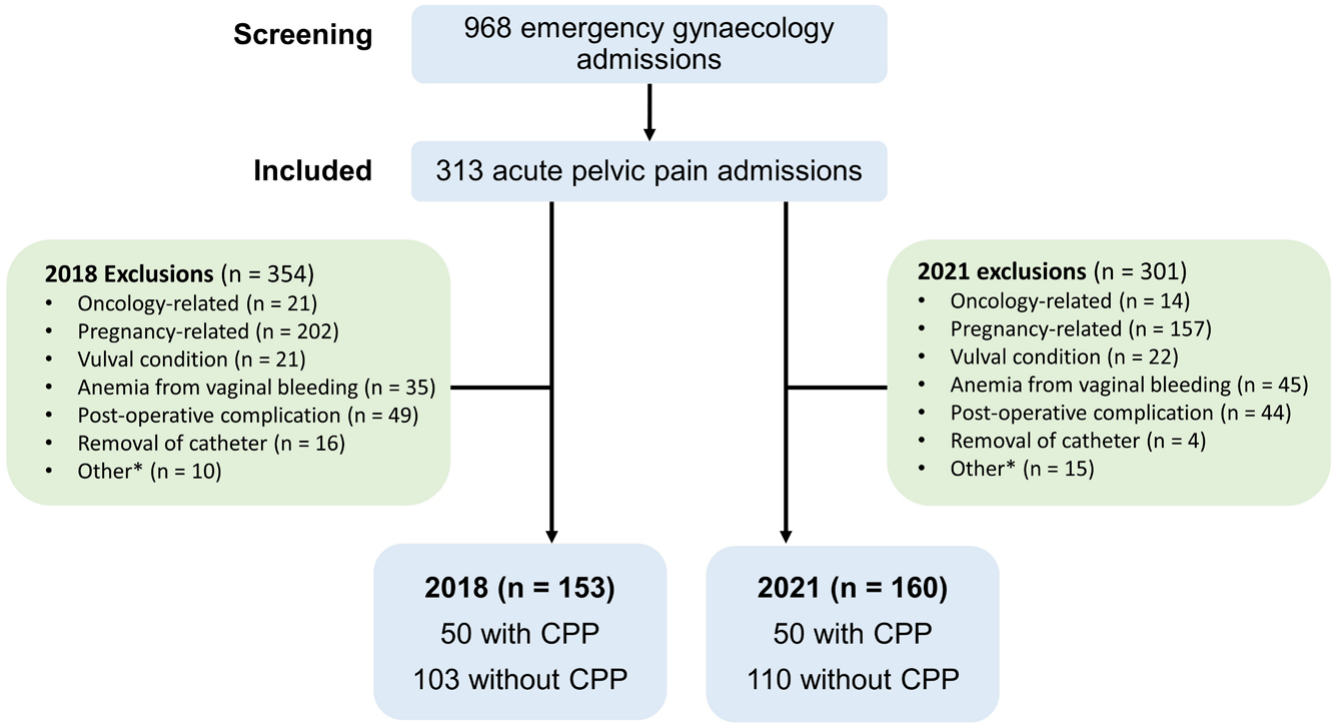
Consort diagram of included and excluded patients. *Other reasons for exclusion include cancelled episode, wrong patient entry, self-harm, cellulitis, biliary colic, overactive bladder, perianal abscess, removal of foreign body, self-harm, not admitted, colorectal case or urine-related issues.

There were 507 and 461 emergency gynaecological admissions identified in 2018 and 2021, respectively. After excluding admissions that did not meet eligibility criteria, 153 and 160 patients were admitted due to acute pelvic pain in 2018 and 2021, respectively (Fig. 1). The mean age of women with a diagnosis of CPP vs women without CPP were similar in both years (*P* = 0.04 and 0.50 in 2018 and 2021, respectively). Women with a diagnosis of CPP were significantly more likely to have had previous acute gynaecological admissions due to pelvic pain than women without CPP (98 and 36%, respectively, in 2018; 94 and 35%, respectively, in 2021; *P* < 0.001 for both years).

There was no significant difference in the number and proportion of women admitted in 2018 and 2021 with a diagnosis of CPP (50/153 and 50/160; *P* = 0.786). Many women with a diagnosis of CPP had endometriosis (48 and 58% in 2018 and 2021, respectively), with superficial peritoneal endometriosis (SPE) as the most common subtype (70 and 52%, respectively) (Supplementary Table 1, see section on supplementary materials given at the end of this article). Of those with SPE, 56 and 50% had previously documented complete surgical excision in 2018 and 2021, respectively.

### Investigations and diagnostic yield

**Figure 2:**
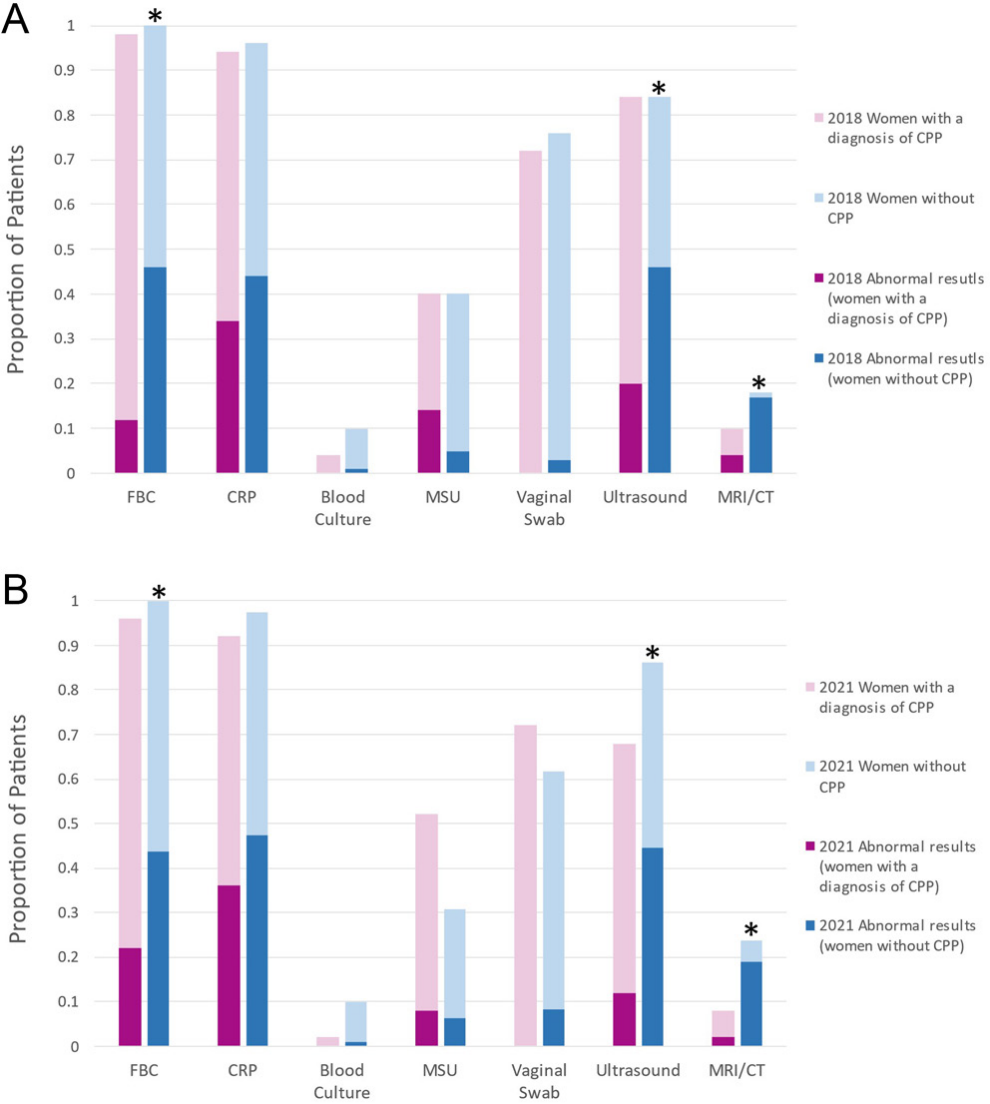
Investigations received and diagnostic yield in 2018 and 2021. CRP, C-reactive protein; CT, computerised tomography; FBC, full blood count; MRI, magnetic resonance imaging; MSU, mid-stream urine culture. The light shaded bars in (A) and (B) show the total proportion of patients who received the listed investigations from July to December in 2018 and 2021, respectively. The darker shaded boxes show the proportion of investigations that yielded new (abnormal) findings. The * represent significantly higher diagnostic yield (*P* < 0.01) in women without CPP compared to women with a diagnosis of CPP.

The types of investigations received and diagnostic yield for women with a diagnosis of CPP in 2018 and 2021 were similar (see Fig. 2). In both years, women with a diagnosis of CPP were significantly more likely to have normal FBC, pelvic ultrasound and CT/MRI findings compared to women without CPP (all *P* ≤ 0.01). Less than 25% of ultrasounds for women with a diagnosis of CPP yielded abnormal results and 0% of VVS cultures were positive. However, 35 and 15% of MSU cultures from women with a diagnosis of CPP were positive in 2018 and 2021, respectively. The diagnosis summary of all acute pelvic pain admissions in both years is summarised in Supplementary Table 2.

### In-hospital management

Women with a diagnosis of CPP rarely required emergency surgical interventions, in contrast to those without a history of CPP (*P* < 0.01, Table 1). COVID-19 did not alter inpatient analgesia and opiate use in women with a diagnosis of CPP. They had significantly greater total opioid use (*P* ≤ 0.001) compared to other pelvic pain admissions, more specifically, oral morphine (*P* < 0.01) and were significantly more likely to require a pain team review (*P* < 0.001).

**Table 1:** In-hospital management summary. Data are presented as *n* (%).

	2018			2021		
	Women with CPP	Women without CPP	*P*-value	Women with CPP	Women without CPP	*P*-value
Total *n*	50	103		50	110	
Simple analgesia	49 (98.0%)	93 (90.3%)	0.083	48 (96.0%)	95 (86.4%)	0.067
Any opiate	38 (76%)	47 (45.6%)	<0.001	38 (76%)	54 (49.1%)	0.001
Oramorph	33 (66%)	42 (40.8%)	<0.01	34 (68%)	46 (41.8%)	<0.01
IV/SC morphine	8 (16%)	18 (17.5%)	0.820	10 (20%)	12 (10.9%)	0.122
Emergency surgery	3 (6%)	28 (27.2%)	0.002	2 (4%)	30 (27.2%)	<0.001
Pain team review	14 (28%)	1 (1.0%)	<0.001	13 (26%)	1 (1.0%)	<0.001
Median days of hospital stay (IQR)	2.0 (1.0–2.8)	1.0 (1.0–3.0)	1.00	1.0 (1.0–2.0)	2.0 (1.0–2.8)	0.700

CPP, chronic pelvic pain; IQR, interquartile range; IV, intravenous; SC, subcutaneous.

### Discharge medication

Overall prescribing patterns of discharge pain medications for women with a diagnosis of CPP remained similar before and after COVID-19 with the exception of the reduction of tramadol prescriptions following the lockdown (Table 2). Women with a diagnosis of CPP were significantly more likely to be prescribed tramadol, oral morphine, tricyclic antidepressants and gabapentinoids for pain control (*P* < 0.01). There was no significant difference in the proportion of patients who started on any type of opioid on discharge regardless of CPP status (Table 3). However, when analysing the specific type of opioid instigated on discharge, women without CPP were more likely to be started on weaker opiates (i.e. dihydrocodeine) while women with a diagnosis of CPP were significantly more likely to be started on stronger opioids such as tramadol and oral morphine for pain management (Table 3).

**Table 2:** Proportion of patients prescribed with analgesia on discharge. In 2018 and 2021, there were six and eight missing discharge prescription letters, respectively, and those patients were excluded from the analysis for discharge outcomes. Data are presented as *n* (%).

	2018			2021		
	Women with CPP	Women without CPP	*P*-value	Women with CPP	Women without CPP	*P*-value
Total *n*	49	98		46	106	
Paracetamol	36 (73.5%)	62 (63.3%)	0.216	32 (69.6%)	77 (72.6%)	0.699
NSAID	16 (32.7%)	33 (33.7%)	0.902	20 (43.5%)	51 (48.1%)	0.599
Any opiate	38 (77.6%)	63 (64.3%)	0.102	30 (65.2%)	64 (60.4%)	0.573
Dihydrocodeine	14 (28.6%	55 (56.1%)	0.0016	22 (47.8%)	55 (51.9%)	0.646
Tramadol	20 (40.8%)	4 (4.1%)	<0.0001	4 (15.2%)	2 (1.9%)	0.0686
Oramorph	12 (24.4%)	4 (4.1%)	0.0004	9 (19.6%)	2 (1.9%)	0.0004
TCAs/duloxetine	11 (22.4%)	0 (0%)	<0.0001	7 (4.3%)	0 (0%)	0.0002
Gabapentinoid	13 (26.5%)	2 (2.0%)	<0.01	7 (15.2%)	0 (0%)	0.0002

CPP, chronic pelvic pain; NSAID, non-steroidal anti-inflammatory; TCA, tricyclic antidepressant.

**Table 3:** Proportion of patients instigated with opioids on discharge. In 2018 and 2021, there were six and eight missing discharge prescription letters, respectively, and those patients were excluded from the analysis for discharge outcomes. Data are presented as *n* (%).

	2018			2021		
	Women with CPP	Women without CPP	*P*-value	Women with CPP	Women without CPP	*P*-value
Total *n*	49	98		46	106	
Any opiate	25 (51.0%)	59 (60.2%)	0.289	23 (50.0%)	63 (59.4%)	0.281
Dihydrocodeine	10 (20.4%)	53 (54.1%)	0.001	16 (34.8%)	52 (49.1%)	0.104
Tramadol	9 (18.4%)	3 (3.1%)	0.0025	2 (4%)	2 (1.9%)	0.585
Oramorph	8 (16.3%)	3 (3.1%)	0.0064	7 (14%)	2 (1.9%)	0.0035

CPP, chronic pelvic pain.

### Cost analysis for women with a diagnosis of CPP

Over the 6 months observational study time period, the median duration and interquartile range of hospital stay for women with a diagnosis of CPP were 1.0 (1.0–2.0) days and 2.0 (1.0–2.8) days in 2018 and 2021, respectively (Table 4). The mean (s.d.) length of hospital stay for women with a diagnosis of CPP were 2.0 (2.0) and 2.0 (2.2) days in 2018 and 2021, respectively. The 6 months total approximate inpatient costs for women with a diagnosis of CPP in 2018 and 2021 were £85,052.08 and £89,578.00, respectively. The equivalent annual total approximate inpatient costs in 2018 and 2021 were, therefore, £170,104 and £179,156, respectively, and there was no significant difference in the mean inpatient hospital cost per patient (*P* = 0.807).

**Table 4:** Cost analysis for women with a diagnosis of CPP. This includes the prices/costs in Great British Pounds (£) from July to December in 2018 and 2021. Costs for full blood count/C-reactive protein/blood culture/mid-stream urine culture/vulvovaginal swabs provided by the Royal Infirmary of Edinburgh laboratory. Pelvic ultrasound, CT and MRI costs from the NHS cost collection.

	Price per unit^†^	2018 (*n* = 50)		2021 (*n* = 50)	
		Cost	*n*	Cost	*n*
Investigation					
Full blood count	£2.86	£140.14	49	£137.28	48
C-reactive protein	£0.89	£41.83	47	£40.94	46
Blood culture	£21.89	£43.78	2	£21.89	1
Mid-stream urine culture	£10.40	£208.00	20	£270.40	26
Dual chlamydia/gonorrhoea vulvovaginal swab	£17.22	£619.92	36	£619.92	36
Transvaginal ultrasound	£817	£34,314.00	42	£27,778.00	34
CT + pre/post contrast	£153	£459.00	3	£612.00	4
MRI + pre/post contrast	£602	£602.00	1	£0	0
Total approximate investigation costs		£36,429		£29,480	
Cost of hospital stay					
Days of hospital stay					
Median (IQR)		2.0 (1.0–2.8)		1.0 (1.0–2.0)	
Mean (S.D.)		2.0 (2.0)		2.0 (2.2)	
Non-malignant gynaecological disorders					
With interventions	£2263.07*	£13,579.23		£18,104.55	
Without interventions	£707.66**	£71,472.85		£71,473.45	
Mean inpatient hospital cost per patient (S.D.)		£1701 (1,516)		£1792 (2,126)	
Total inpatient costs					
July–December		£85,052.08		£89,578.00	
Annual equivalent		£170,104.16		£179,156.00	

^†^Investigation costs represent the number of patients receiving each investigation during their admission.

*HRG code: MB09C. Currency description: non-malignant gynaecological disorders with interventions, with CC score 0–2. Cost is per inpatient bed day.

**HRG code: MB09F. Currency description: non-malignant gynaecological disorders without interventions, with CC score 0–2. Cost is per inpatient bed day.

CT, computerised tomography; MRI, magnetic resonance imaging; s.d., standard deviation.

When analysing the ‘pure cost of investigations’ separately from the ‘total inpatient costs’, the total approximate costs of investigations from July to December in 2018 and 2021 for women with CPP were £36,429 and £29,480, respectively (Table 4). The costliest investigations were transvaginal ultrasounds (£34,314.00 and £27,778.00 in 2018 and 2021, respectively) followed by combined gonorrhoea/chlamydia VVS (£619.92 in both years).

## Discussion

This study found that there was no significant difference in the number of emergency admissions to gynaecology for pain flares before and after COVID-19 in women with previously diagnosed CPP. When compared to admissions for women without CPP, women with a diagnosis of CPP required significantly more opioids for pain control and had undergone investigations that were more likely to yield normal results.

In the Royal College of Obstetricians and Gynaecologists ‘Restoration and Recovery’ report published in April 2021 after the initial COVID-19 lockdowns, benign gynaecology admissions due to acute pelvic pain refractory to simple analgesia remained an indication for emergency outpatient assessment (RCOG 2021). We originally hypothesised a surge in emergency admissions for CPP flares after COVID-19 due to the backlog in access to elective gynaecology services, but this was not observed. During the initial lockdowns in 2020, observational studies reported a 12–35% decrease in all emergency department attendance which may have been explained by avoidance behaviours with fears of contracting the virus (Czeisler *et al.* 2020, Vollmer *et al.* 2021). These avoidance behaviours may have continued after lockdown restrictions were eased and explain why the number of CPP flare admissions was lower than expected.

On the contrary, improved pain control may also explain the similar number of CPP patients presenting to acute admissions observed. During the lockdown, initiatives from several university websites and advocacy organisations on social media disseminated self-management strategies for CPP and endometriosis (Leonardi *et al.* 2020*b*). These efforts aimed to increase awareness of various problem-focused strategies (e.g. managing work and study, social support, sleep, physical exercise) as adjunctive or alternative solutions for pain management which may have been successful in reducing pelvic pain flares after the lockdown.

To our knowledge, this is the first study to compare inpatient management of pelvic pain flares before and after the COVID-19 lockdown in women with a diagnosis of CPP and perform a health economics analysis to explore their healthcare resource use. Missing data were minimal in this retrospective cohort study. Restriction of data analysis to a single hospital limits the generalisability of results.

Whilst this study did not find a difference in the number of emergency admissions for pelvic pain exacerbations in women with a diagnosis of CPP before and after the COVID-19 pandemic, it does highlight the ongoing challenges with managing CPP and associated pain flares. We found that 98 and 94% of women with a diagnosis of CPP presenting with a pelvic pain flare in 2018 and 2021, respectively, were previously admitted to gynaecology for acute pelvic pain exacerbations. When analysing analgesia prescribing patterns, it is evident that opioids for pain control during in-hospital management, and more concerningly, on discharge, continue to be significantly higher compared to women without CPP. Previous studies in the United States have shown that early opioid prescribing for opioid-naïve patients is associated with an increased risk of chronic dependence (Deyo *et al.* 2017, Shah *et al.* 2017). The harms of long-term opioid use have been well-documented including the risks of drug abuse, overdose, chronic constipation and serious cardiovascular events (Von Korff *et al.* 2011). However, there is insufficient evidence to support the effectiveness of long-term opioid therapy for all forms of chronic pain including CPP (Chou *et al.* 2015). Several international guidelines have been published discouraging the use of long-term opioids for managing CPP and more work is needed to optimise safe and effective analgesia prescriptions for women with CPP (RCOG 2012, Dowell *et al.* 2016, AAGL 2017, NICE 2021). Ongoing improvements in managing CPP in the community may also help reduce repeat acute admissions due to pain flares (Alderwick & Dixon 2019).

The diagnostic yield of investigations for women with CPP was low in both years, but it remains challenging to achieve the correct balance between over-investigation (and the cost thereof), against the risk of missing pathology with both short and long-term consequences. The costs of investigations over the 6-month periods were calculated based on whether a patient did or did not have the investigation rather than the total number of investigations each patient received as this data was not extracted. Although this may underestimate the costs for some investigations (e.g. FBC and CRP) that may be repeated during admission, investigations that represent proportionally the largest group of costs (e.g. pelvic ultrasounds, combined VVS and CT/MRI scans) are usually performed once during a patient’s hospital admission and are more likely to reflect typical resource use quantities in the CPP population. All swabs for chlamydia and gonorrhoea were negative and would have cost the NHS approximately £1239.84 each year, but a missed diagnosis of pelvic inflammatory disease may have long-term consequences for fertility. However, since women with a diagnosis of CPP are significantly more likely to have previous emergency admissions due to pelvic pain, repeated investigations with little diagnostic yield can add to the overall costs. Therefore, clinicians should query the value of combined chlamydia/gonorrhoea swabs in women with a diagnosis of CPP without additional risk factors. An exception to the rationalisation of microscopy would be urine culture, as infection appears to be a precipitant for pelvic pain flares.

## Conclusion

Overall, there was no significant difference observed in the proportion of women with a diagnosis of CPP admitted for pelvic pain flares before and after the COVID-19 lockdown period. Clinicians should have a higher index of suspicion for urinary tract infections in women with CPP experiencing pain flares. On the contrary, dual VVS for chlamydia and gonorrhoea have little diagnostic value in women with CPP without additional risk factors. Women with a diagnosis of CPP often require more opioids for in-hospital pain management and are more likely to be started on strong opioids on discharge. Continuous efforts are needed to improve long-term pain management and optimise resource utilisation.

## Supplementary Materials

Supplementary Table 1. CPP characteristics on admission

Supplementary Table 2. Diagnosis summary of all acute pelvic pain admissions

## Declaration of interest

AH’s institution (University of Edinburgh) has received payment for consultancy and grant funding from Roche Diagnostics to assist in the early development of a possible blood diagnostic biomarker for endometriosis. AH has received grant funding from the MRC and NIHR for endometriosis research. AH is co-editor in chief of *Reproduction and Fertility* and was not involved in the review or editorial process associated with this paper. LW has received grant funding from the NIHR and Roche Diagnostics.

## Funding

KK was supported by the INSPIRE undergraduate research program (supported by the Wellcome Trusthttp://dx.doi.org/10.13039/100010269). The funders did not play a role in the study design, data collection and analysis, or writing of the manuscript. AH and LW were supported by a grant from the Medical Research Councilhttp://dx.doi.org/10.13039/501100000265 (MR/N022556/1).

## Author contribution statement

LHR Whitaker and A Horne designed the study. K Kuan extracted data, performed the data analysis and drafted the original manuscript. A Neilson assisted with cost analysis. All authors edited, reviewed and approved the final manuscript.
