# Supplementary material for: Comparing inpatient management of chronic pelvic pain flares before and after the COVID-19 pandemic
Source: Reprod Fertil. 2023 Jun 16;4(2):e230004. doi: 10.1530/RAF-23-0004 (PMC10305628; doi:10.1530/RAF-23-0004)
Supplement: Supplementary Table 1. CPP characteristics on admission [file supplementary_table_1.pdf]

Supplementary Table 1. CPP characteristics on admission

	2018	2021
Women with diagnosed CPP (total)	50	50
CPP with laparoscopy demonstrating no obvious pelvic pathology	11	6
CPP with no diagnostic laparoscopy (no known pelvic pathology)	15	15
CPP with endometriosis (total)	24	29
Excised DE	2	3
Unexcised DE	1	4
Excised SPE	9	10
Unexcised SPE	5	5
Ovarian endometrioma alone	0	1
Excised endometrioma alone	1	0
Endometriosis subtype not stated	6	6

Abbreviations: CPP, chronic pelvic pain; DE, deep endometriosis; SPE, superficial peritoneal endometriosis
