# Supplementary material for: Comparing inpatient management of chronic pelvic pain flares before and after the COVID-19 pandemic
Source: Reprod Fertil. 2023 Jun 16;4(2):e230004. doi: 10.1530/RAF-23-0004 (PMC10305628; doi:10.1530/RAF-23-0004)
Supplement: Supplementary Table 2. Diagnosis summary of all acute pelvic pain admissions [file supplementary_table_2.pdf]

Supplementary Table 2. Diagnosis summary of all acute pelvic pain admissions

	2018 (%, n)	2021 (%, n)
Definitive Diagnosis		
Ovarian cyst-related	19.0% (29)	19.4% (31)
Appendicitis	2.6% (4)	0.6% (1)
Diverticulitis	1.3% (2)	0.0% (0)
Hydro/pyosalpinx	1.3% (2)	2.5% (4)
Pelvic inflammatory disease	1.3% (2)	3.1% (5)
Renal-related	1.3% (2)	0.6% (1)
Urinary tract infection	0.7% (1)	1.3% (2)
Other	7.2% (11)[1]	4.4% (7)[4]
Suspected Diagnosis		
Chronic pelvic pain flare	25.5% (39)	26.9% (43)
Non-specific abdominal pain*	19.0% (29)	15.0% (23)
Ovarian cyst related	7.2% (11)	13.1% (21)
Pelvic inflammatory disease	2.6% (4)	3.8% (6)
Mittelschmerz	2.0% (3)	1.9% (3)
Hydrosalpinx	2.0% (3)	0%
Urinary tract infection	0.7% (1)	1.3% (2)
Thrush	0.7% (1)	0% (0)
Other	0.7% (1)[2]	1.3% (2)[5]
Uncertain	5.2% (8)[3]	5.0% (8)[3]
Total number of patients	153	160

Diagnosis summary of all acute pelvic pain admissions in 2018 and 2021 (regardless of chronic pelvic pain history).

[*]Non-specific abdominal pain = no cause for pain found and no prior history of chronic pelvic pain and/or endometriosis
[1]Haematometra, haematocolpos, mesenteric adenosis, endometrial polyp, osteopubis, constipation, sepsis, pelvic adhesions, fibroma, fibroid
[2]Lower respiratory tract infection
[3]Multiple suspected diagnoses
[4]Fibroid, rectus abdominus haematoma, dysmenorrhoea, chlamydia, septicaemia, adenomyosis, constipation
[5]Adenomyosis, constipation
